# A comparison of misoprostol vaginal insert and misoprostol vaginal tablets for induction of labor in nulliparous women: a retrospective cohort study

**DOI:** 10.1186/s12884-017-1647-3

**Published:** 2018-01-05

**Authors:** Kjersti Engen Marsdal, Ingvil Krarup Sørbye, Lise C. Gaudernack, Mirjam Lukasse

**Affiliations:** 1 Department of Obstetrics, Oslo University Hospital Rikshospitalet, P.O. Box 4956 Nydalen, 0424 Oslo, Norway; 2 Oslo and Akershus University College, Faculty of Health Sciences, Department of Nursing and Health Promotion, P.O. Box 4, 0130 Oslo, Norway

**Keywords:** Labor induction, Cervical ripening, Misoprostol, Nulliparity, Cesarean section

## Abstract

**Background:**

Since Misoprostol Vaginal Insert (MVI - Misodel ®) was approved for labor induction in Europe in 2013, to date, no study has been published comparing MVI to Misoprostol vaginal tablets (MVT). The aim of this study, performed as part of a quality improvement project, was to compare the efficacy and safety of 200 μg MVI versus 25 μg MVT for labor induction in nulliparous women.

**Methods:**

This retrospective cohort study included 171 nulliparous singleton term deliveries induced with MVI (*n* = 85) versus MVT (*n* = 86) at Oslo University Hospital Rikshospitalet, Norway, from November 2014 to December 2015. Primary outcomes were time from drug administration to delivery in hours and minutes and the rate of cesarean section (CS). Results were adjusted for Bishop Score and pre-induction with balloon catheter.

**Results:**

Median time from drug administration to delivery was shorter in the MVI group compared to the MVT group (15 h 43 min versus 19 h 37 min, *p* = 0.011). Adjusted for confounding factors, mean difference was 6 h 3 min (*p* = 0.002). The risk of CS was 67% lower in the MVI group compared to the MVT group (11.8% versus 23.3%, OR = 0.33; adjusted 95% CI 0.13–0.81). Adverse neonatal outcomes did not differ between the groups.

**Conclusions:**

In a setting of routine obstetric care, MVI seems to be a more efficient labor induction agent than MVT, and with a lower CS rate and no increase in adverse infant outcomes.

## Background

Induction of labor is one of the most frequently performed obstetrical interventions. The decision to induce labor is made if ending the pregnancy is considered more beneficial for the mother or the baby than awaiting spontaneous onset of labor. Induction of labor has increased over the last decades across Europe. In 2010, in 15 of 25 countries in Europe, more than 20% of the labors were induced [1]. In Norway, the induction rate increased from 12.5% in 2003 to 20.3% in 2013. The most common indications for induction of labor were pre-labor rupture of the membranes (PROM) and post-term pregnancy [2].

Whereas induction of multiparous women has a high success rate, the induction of nulliparous women poses a particular obstetrical problem. Inductions in nulliparous women with an unfavorable or unripe cervix carry an increased risk of dystocia and protracted labor [3, 4]. Conversely, induction of labor also poses a risk of uterine tachysystole and subsequent fetal distress [5]. Protracted labor and fetal distress are the two main indications for CS in Norway [6]. CS in the first delivery also has consequences for subsequent labors, as the repeat CS rate in Norway is 50% [6]. Thus it is of clinical importance to determine the safety and efficacy of new methods for induction of labor for nulliparous women in particular.

Misoprostol is a synthetic prostaglandin E1 analog and has been used off-label for cervical ripening and labor induction since the 1980s [7]. For labor induction in women with an unfavorable cervix, Misoprostol is more effective than other methods such as oxytocin, Dinoprostone and placebo, with no differences in adverse perinatal or maternal outcomes [7]. In Norway, Misoprostol 25 μg tablets administered vaginally every 4–6 h, has been the most commonly used method for inducing labor with an unfavorable cervix [2].

In 2013 a 200 μg Misoprostol vaginal insert (MVI – Misodel ®) received approval in Europe [8, 9]. In a phase III trial, MVI was compared to a Dinoprostone vaginal insert, a prostaglandin E2 analog. This trial reported significantly reduced times to delivery and no evidence of differences in maternal or neonatal safety outcomes [10]. Since the phase III trial, only three studies have compared MVI to other induction methods in terms of delivery outcomes [11–13]. Neither of these studies have presented data from nulliparous women exclusively, and no studies have compared MVI with MVT.

In a daily obstetric practice, individual care might lead to deviation from protocol. Thus, results from experimental studies are not always valid for obstetric care. The aim of the present study was to compare efficiency and safety of MVI versus MVT for labor induction in nulliparous women within a routine care setting. Our primary outcomes were time from drug administration to delivery and the rate of CS.

## Methods

During 2014–2015 a national obstetric quality improvement project on CS was launched in Norway. One of the preselected focus areas was induction of labor in nulliparous women. During this period, our obstetrical unit improved our protocols for selecting women for induction of labor and improved adherence to the protocols for the induction procedures. As part of the project, MVI was introduced as an alternative to MVT in nulliparous women from November 2014 onwards.

In this study we included induced nulliparous women that delivered at Oslo University Hospital Rikshospitalet, Norway, from November 2014 through December 2015. The unit is a tertiary obstetrical unit with around 2800 deliveries annually. Women were included if their labors were induced with MVI or MVT, if they had no previous uterine surgery or other uterine abnormality and gave birth to a single fetus, in cephalic presentation, at gestational age of 37 weeks or more. This corresponds to Robson group 2a in the 10-group classification system [14]. As the study was a part of a quality improvement project conducted within a routine care setting, no randomization was performed. Both MVI and MVT were used for induction of labor during the whole study period and the choice of method was decided usually jointly by the obstetric consultant and the midwife on call. During the study period, 174 nulliparous women were initially included. Of these, three women, who received MVT after the MVI was accidentally removed, were excluded from the study.

**Fig. 1:**
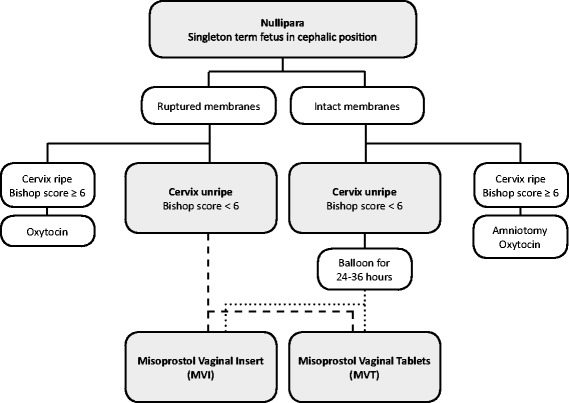
Flow chart of the protocol for induction of labor in nulliparous women

In Norway, there is no standardized protocol for induction of labor. The department’s protocol for induction of labor in nulliparous women during the study period is presented in Fig. 1.

MVI is a removable vaginal insert with a reservoir of 200 μg Misoprostol, released at a mean rate of approximately 7 μg per hour over a period of 24 h. MVI was inserted once, while the insertion of a 25 μg MVT was repeated every 4 h. After insertion of MVI or MVT, the women remained in bed with continuous cardiotocography (CTG) for one hour. A CTG was performed every 4–6 h; once regular contractions were established or according to the department’s procedures. In women with a non-reassuring CTG during the induction process, the insert was withdrawn or tablet removal was attempted. The MVI was removed when the midwife considered that labor was established; if the CTG showed a non-reassuring pattern or if the 24-h dosing period was completed. For women not in labor after the dosing period, artificial rupture of membranes was performed, followed by oxytocin infusion. The oxytocin infusion was started at 5 mU/min and increased by 5 mU/min every 30 min until adequate uterine activity, defined as 4–5 contractions per 10 min. The maximum infusion rate was 30 mU/min. The oxytocin infusion was stopped or decreased if the woman had more than 5 contractions per 10 min. For inductions with MVT, artificial rupture of the membranes was performed when the Bishop Score reached 6 or more, followed by oxytocin infusion as described. At the end of day 3 or beginning of day 4, artificial rupture of the membranes was attempted even if the Bishop Score was below 6. The administration of MVT could be postponed if the woman was having regular contractions, if the woman needed to rest at night due to a long induction process and in rare cases due to logistic considerations. Inductions could be started any time during the day. No progress of labor and failed induction was according to the departments protocol defined as no progression after 6 h with the maximum dose of oxytocin. Individual assessments on labor progression were made by the obstetric consultant on call.

Our primary outcome regarding efficiency was time from drug administration to delivery. Secondary outcomes included time from drug administration to onset of the active phase of labor and labor duration. As to safety, our primary outcome was the rate of CS. Secondary outcomes included the proportion of operative and spontaneous vaginal deliveries, the use of oxytocin stimulation, the proportion of Apgar Score < 7 after 5 min and the rate of CS and operative vaginal deliveries due to fetal distress. All CTG-registrations from labors ending with operative delivery due to fetal distress were investigated for uterine tachysystole, defined as >5 contractions in 10 min.

Labor onset was defined as when the partogram was started by the attending midwife. Onset of active phase of labor was defined as regular, painful contractions that led to a change in the cervix. PROM was defined as ruptured membranes without contractions. In this group, labor was induced after 24–48 h, and signs of infection were monitored until delivery. In women with PROM and meconium-stained amnion fluid, induction was started without delay.

### Statistical analysis

Maternal characteristics and indications for labor induction were compared between the two groups using Student’s t-test for continuous variables and chi-square test for dichotomous variables. For all outcomes, we identified potential confounding variables a priori according to previous knowledge of factors that could affect the likelihood of successful induction of labor using MVI or MVT [15–18]. Potential confounding factors included maternal age, body mass index, gestational age, birth weight, Bishop Score, PROM and pre-induction with balloon catheter. As a higher proportion of women in the MVI groups were induced due to hypertension/preeclampsia, the reason for induction was also considered a potential confounding factor. True confounders were defined as confounders that changed the results with more than 10%. For time outcomes, we used Student’s t-test and Mann-Whitney U test to compare the two groups. Bishop Score and balloon catheter were identified as true confounding factors and were included in linear regression models with the forced entry method for the time outcomes. Due to skewed distributions, we also performed analyses with log transformed time variables in the model. To evaluate if labors interrupted by CS influenced the results, we also performed Cox regression with log rank test, censoring CS and with adjustment for Bishop Score and pre-induction with balloon catheter. In terms of delivery mode outcomes, we calculated crude and adjusted odds ratios (OR) with 95% confidence intervals (CI) in logistic regression models. In these analyses Bishop Score and balloon catheter were identified as true confounding factors and included in the models. To evaluate if fetal distress led to more operative deliveries in one of the groups, the proportion of CS and operative vaginal deliveries due to fetal distress were compared between the groups. Less than 2% of the data were missing. Missing data were excluded pairwise. A *p*-value of <0.05 was considered to indicate statistical significance. Statistical analyses were performed with IBM SPSS Statistics for Windows, Version 21 Armonk, NY: IBM Corp.

### Ethical considerations

The study was approved by the Oslo University Hospital Data Protection Official for Research (2012/9668). The study was also evaluated by the Regional Committee for medical and health research ethics (REC South East in Norway); however, as the study was limited to observations during standard clinical care, written informed consent was waivered.

## Results

A total of 171 women were included in the study. Of these, 85 (49.7%) received MVI and 86 (50.3%) received MVT. Maternal and pregnancy characteristics were comparable in the two groups except for the mean Bishop Score, which was lower in the MVI group (Table 1).

**Table 1:** Maternal characteristics and indications for labor induction in nulliparous women induced with Misoprostol Vaginal Insert (MVI) compared to Misoprostol Vaginal Tablets (MVT)

	MVI, *n* = 85		MVT, *n* = 86		
	mean or n	SD or %	mean or n	SD or %	*p*-value
Maternal age in years^a^	32.5	4.8	32.9	6.0	0.64
Body Mass Index^b^	24.6	5.5	24.4	4.8	0.807
Gestational age in days^a^	281	9.6	282	9.8	0.393
Bishop score^c^	3.1	1.2	3.6	1.5	0.016
Birthweight	3454	484	3485	520	0.692
Preinduction with balloon catheter	44	51.8	40	47.1	0.645
Primary indication for induction					
- Pre labor ruptures of membranes (PROM)	21	24.7	23	27.1	0.861
- Preeclampsia/hypertension	25	29.4	12	14.1	0.026
- Fetal concerns	16	18.8	18	21.2	0.848
- Postterm pregnancy^d^	8	9.4	13	15.3	0.351
- Maternal concerns	10	11.8	9	10.6	1.000
- Other	5	5.9	10	11.6	0.279

^a^At date of delivery

^b^From first prenatal visit

^c^At insertion of MVI or first MVT

^d^≥42 + 0 weeks, ≥41 + 2 if maternal age ≥ 40 years

As for the primary indication for induction, more women in the MVI group were induced due to preeclampsia/hypertension than in the MVT group, whereas other indications were similarly distributed.

**Fig. 2:**
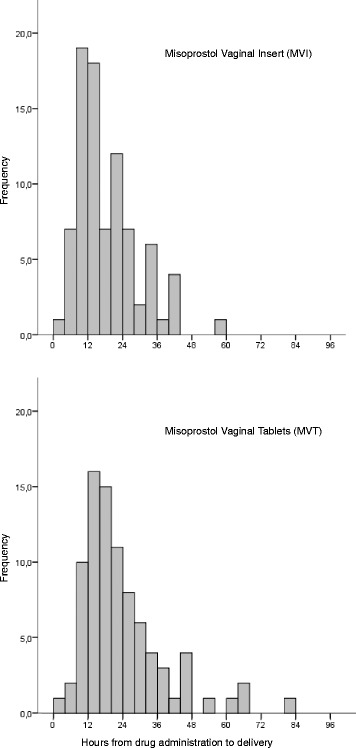
Time from drug administration to delivery in women induced with Misoprostol Vaginal Insert (MVI) (*n* = 85) compared to Misoprostol Vaginal Tablets (MVT) (*n* = 86)

The time interval from drug administration to delivery showed a skewed distribution with a right tail in both the MVI and the MVT groups (Fig. 2).

The average time from drug administration to delivery was significantly shorter in the MVI group compared to the MVT group (median time 15 h 43 min versus 19 h 37 min, *p* = 0.011), see Table 2. In regression models adjusting for Bishop Score and pre-induction with balloon catheter, the mean difference was 6 h 3 min (*p* = 0.002). The result did not change in models with log transformed outcomes (*p* = 0.001, data not shown). We conducted sensitivity analyses where we adjusted for additional potential confounders; however, the results did not change.

**Table 2:** Time outcomes in nulliparous women induced with Misoprostol Vaginal Insert (MVI) compared to Misoprostol Vaginal Tablets (MVT)

	MVI	MVT	Difference	*p*-value
Time intervals	*n* = 85	*n* = 86		
Time from drug administration to delivery				
Median (IQR)	15:43 (12:29)	19:37 (14:30)	3:54	0.011
Crude mean (SD)	18:39 (10:23)	23:42 (14:29)	5:03	0.010
Adjusted mean difference (CI; 95%)^a^			6:03 (2:20–9:46)	0.002
Time from drug administration to onset of active labor				
Median (IQR)	10:37 (10:42)	11:28 (12:13)	0:51	0.215
Crude mean (SD)	13:04 (8:32)	16:20 (13:27)	3:16	0.061
Adjusted mean difference (CI; 95%)^a^			4:16 (0:54–7:38)	0.013
Time from onset of active labor to delivery				
Median (IQR)	4:06 (6:54)	6:46 (5:50)	2:40	0.002
Crude mean (SD)	5:35 (4:36)	7:22 (4:09)	1:47	0.009
Adjusted mean difference (CI; 95%)^a^			1:47 (0:28–3:06)	0.008

Presented as hours:minutes

^a^Adjusted for Bishop Score and pre-induction with balloon catheter

**Fig. 3:**
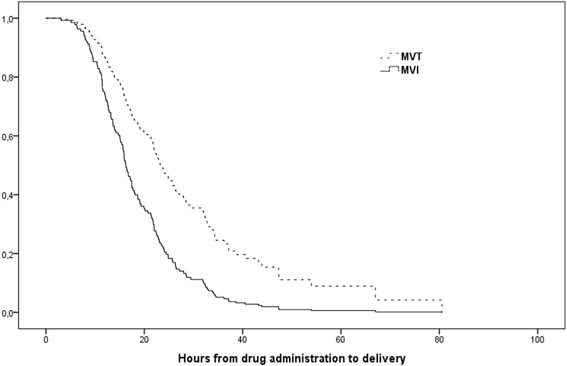
Survival plot for time from drug administration to delivery in nulliparous women induced with Misoprostol vaginal insert (MVI) compared to Misoprostol vaginal tablets (MVT)

In the Cox model, where we censored deliveries interrupted by CS, the hazard ratio was increased by a factor of 2.1 in the MVI group compared to the MVT group, thus confirming the shorter time interval from drug administration to delivery in the former (see Fig. 3 and Table 3). In 9 women, the inductions were started in the evening and the 2nd dose of MVT was delayed so that the woman could rest at night. Excluding these women from the analyses did not change the results for the primary outcomes; time from drug administration to delivery and rate of CS.

**Table 3:** Adjusted hazard ratio in relation to time from drug administration to delivery in nulliparous women induced with Misoprostol Vaginal Insert (MVI) compared to Misoprostol Vaginal Tablets (MVT)

	Adjusted Hazard ratio	95% CI	*p*-value*
Misoprostol vaginal insert (MVI)	2.11	1.48–3.02	0.001
Misoprostol vaginal tablets (MVT)	1	Reference	Reference

*From Log rank test

As to delivery mode, women induced with MVI were less likely to be delivered by CS, compared to those induced with MVT (11.8% versus 23.3%), see Table 4. In models adjusting for Bishop Score and pre-induction with balloon catheter, there was a 67% reduced risk of CS in the MVI group compared to the MVT group (adjusted OR 0.33; 95% CI 0.13–0.81, *p* = 0.016). The results did not change in sensitivity analyses where we stratified for other potential confounders.

**Table 4:** Delivery mode in nulliparous women induced with Misoprostol Vaginal Insert (MVI) compared to Misoprostol Vaginal Tablets (MVT)

	MVI, *n* = 85		MVT, *n* = 86		Crude, *n* = 171			Adjusted^a^, *n*=169^b^		
	*n*	%	*n*	%	OR	95% CI	*p*-value	OR	95% CI	*p*-value
Cesarean section	10	11.8	20	23.3	0.44	0.19–1.01	0.052	0.33	0.13–0.81	0.016
Operative vaginal delivery	19	22.4	18	20.9	1.09	0.53–2.25	0.821	1.14	0.54–2.39	0.733
Spontaneous vaginal delivery	56	65.9	48	55.8	1.45	0.78–2.69	0.236	1.60	0.84–3.04	0.150

^a^Adjusted for Bishop score and pre-induction with balloon catheter

^b^2 excluded due to missing data on Bishop score

We found no difference in the rate of women delivered by CS due to fetal distress in the MVI versus the MVT groups; however, numbers were few (*n* = 5 (5.9%) versus *n* = 8 (9.3%)). Similarly we found no difference in the proportion of operative vaginal deliveries due to fetal distress in the two groups (MVI: n = 8 (9.4%) versus MVT: *n* = 11 (12.8%)). The number of labors diagnosed with uterine tachysystole and that ended with operative delivery due to fetal distress were few in both groups (MVI *n* = 4, MVT *n* = 6). Three neonates had an Apgar Score < 7 after 5 min; two in the MVI group and one in the MVT group. None of the neonates were diagnosed with metabolic acidosis (defined as umbilical artery pH <7.00 and/or base deficit ≥12). Forty five (53%) women in the MVI group needed oxytocin during labor, compared to 63 (73%) in the MVT group (*p* = 0.006).

## Discussion

In this study, in a setting of routine obstetric care, induction of labor with MVI was associated with a shorter time from drug administration to delivery compared to MVT. Both time from drug administration to onset of active labor and labor duration were shorter in the MVI group compared to the MVT group. Women induced with MVI had a lower risk of CS compared to women induced with MVT. There were no differences between the groups for the proportion of operative deliveries due to fetal distress or Apgar Score < 7 after 5 min.

To our knowledge, this is the first study comparing MVI to MVT. After the European approval on MVI, three studies comparing MVI other induction methods have been published [11–13]. Two studies comparing MVI to Dinoprostone insert [11, 13] did not find the same benefits for MVI over Dinoprostone insert as the phase III trial [10]. One study comparing MVI to Oral Misoprostol found shorter time from drug administration to delivery and a higher CS rate in the MVI group compared to Oral Misoprostol [12]. However, numbers for nulliparous women were not reported separately in any of the studies. Given the strong predictive value of parity on successful induction of labor, the results cannot directly be compared to our findings [15, 17].

We regard the mean adjusted difference in time from drug administration to delivery of 6 h 3 min as of clinical relevance, demonstrating MVI as the most effective induction agent. One contributory factor to the differences in efficiency could be deviation from the protocol in the MVT group, compared to the MVI group. Compliance with procedure is more likely with the MVI as non-compliance would require removal of the insert, while non-compliance with the MVT is “just waiting a bit” with the next tablet. As prolonged latency and labor might lead to an increased risk of emergency CS and adverse maternal and neonatal outcome, an induction agent that is effective in the everyday routines of the department is preferable [6, 19, 20]. On the other hand, uterine tachysystole is a matter of considerable safety concern, especially for the fetus, when inducing labor [5]. The 5 min Apgar Score < 7 did not differ between the groups, neither did the proportion of operative deliveries due to fetal distress; however, cases were few. The lower rate of CS does however suggest that MVI is a safer alternative for the mother compared to MVT.

Compared to other studies, women in our study, both in the MVI and in the MVT groups, had more efficient induction processes and a lower CS rate. The median time from drug administration to delivery of 15.7 h in the MVI group is considerably shorter than the 25.9 h and 29.2 h reported on nulliparous women induced with MVI in previous studies. However, in these studies MVI was compared to Dinoprostone [10, 21]. Previous studies of 25 μg MVT have also reported longer drug administration to delivery intervals compared to our findings, also in nulliparous women [22–24]. Median time has been reported to be 23.0 h [22], compared to 19.6 h in our study, and mean time 28.0 and 28.2 h [23, 24], compared to 23.7 h. In terms of CS, the rate found in our study is lower compared to previous studies on nulliparous women. In the MVI group, 11.8% underwent a CS in our study, compared to 32.9% and 34.5% in previous studies [10, 21]. For MVT, previous studies show more divergent results, from 20% to 42% [22, 25], compared to our result of 23.3%. These differences in efficiency and CS rate between the cited studies and our study may reflect provider-preference and a differential induction- and labor management policy. The overall CS rate in Norway and Scandinavia is low compared to other high income countries. In 2014, 16.6% were delivered by CS in Norway, compared to 32.2% in USA and 26.2% in UK [26–28]. A high proportion of pre-induction with balloon catheter (49.1%) and PROM (25.7%) might have contributed to increased efficiency and lower CS rate.

This study has several limitations. First, this retrospective cohort study was a part of a quality improvement project, focusing on induction in nulliparous women. Thus, there was no attempt of randomization. One could hypothesize that high risk pregnancies more often were induced using MVT, especially in the beginning of the study period, as the doctors and midwives were more familiar with this method. This might have contributed to a higher CS rate in the MVT group. However, as the maternal characteristics and the indications for induction of labor did not show major differences, this selection bias is unlikely to have had a major impact on our results. Conversely, the average Bishop Score was higher in the MVT group, which should correspond to more favorable cervix status. Furthermore fewer women had preeclampsia/hypertension in the MVT group which is a known risk factor for CS [29]. Although the lack of randomization could be considered a weakness, the results reflect what happened when MVI was introduced to a maternity department, without any adjustments in the treatment due to research considerations. Our results were robust across several different statistical models. Second, labor onset in this study was defined as when the midwife present defined active labor and started the partogram. This subjective assessment will differ between midwives. However, this is unlikely to represent a differential bias and is unlikely to influence the time from drug administration to delivery. Finally, the women in our study were not asked about their birth experience. As a negative birth experience is associated with an increased risk of postpartum depression, subsequent fear of childbirth and request for elective CS [6, 30], this would have added valuable information to the study.

## Conclusions

In this study among nulliparous women in a routine care setting we found 200 μg MVI to be a more efficient and safe labor induction agent compared to 25 μg MVT. The time from drug administration to delivery was significantly shorter and the CS rate reduced in women induced with MVI, compared with MVT. Future studies comparing methods for labor induction should acknowledge the particular status of nulliparous women.

## Abbreviations

CI: Confidence interval
CS: Cesarean section
CTG: Cardio-tocographic monitoring
MVI: Misoprostol vaginal insert
MVT: Misoprostol vaginal tablet
OR: Odds ratio
PROM: Pre-labor rupture of the membranes
SD: Standard deviation

## References

[1] European perinatal health report: Health and Care of Pregnant Women and Babies in Europe in 2010. Euro-Peristat. http://www.europeristat.com/reports/european-perinatal-health-report-2010.html. Accessed 03 Sept 2016.

[2] Dögl M, Vanky E, Heimstad R (2016). Changes in induction methods have not influenced cesarean section rates among women with induced labor. Acta Obstet Gynecol Scand.

[3] Yeast JD, Jones A, Poskin M (1999). Induction of labor and the relationship to cesarean delivery: a review of 7001 consecutive inductions. Am J Obstet Gynecol.

[4] Vahratian A, Zhang J, Troendle JF, Sciscione AC, Hoffman MK (2005). Labor progression and risk of cesarean delivery in electively induced nulliparas. Obstet Gynecol.

[5] Stewart RD, Bleich AT, Lo JY, Alexander JM, McIntire DD, Leveno KJ (2012). Defining uterine tachysystole: how much is too much?. Am J Obstet Gynecol.

[6] Kolås T, Hofoss D, Daltveit AK, Nilsen ST, Henriksen T, Häger R, Ingemarsson I, Øian P (2003). Indications for cesarean deliveries in Norway. Am J Obstet Gynecol.

[7] Hofmeyr GJ, Gülmezoglu AM, Pileggi C. Vaginal misoprostol for cervical ripening and induction of labour. Cochrane Database Syst Rev. 2010;10 10.1002/14651858.CD000941.pub2 PMC7061246 20927722

[8] Misodel Summary of Product Characteristics. https://www.ferring.com/en/media/press-releases/2013/misodel-17oct13/. Accessed 01 Oct 2016.

[9] Heads of Medicines Agencies. Misoprostol gynaecological indication PSUR SAR. 2015. http://www.hma.eu/search.html?id=6&q=misodel&L=0. Accessed 28 Sept 2016.

[10] Wing DA, Brown R, Plante LA, Miller H, Rugarn O, Powers BL (2013). Misoprostol vaginal insert and time to vaginal delivery: a randomized controlled trial. Obstet Gynecol.

[11] Mayer RB, Oppelt P, Shebl O, Pömer J, Allerstorfer C, Weiss C (2016). Initial clinical experience with a misoprostol vaginal insert in comparison with a dinoprostone insert for inducing labor. Eur J Obstet Gynecol Reprod Biol.

[12] Dobert M, Brandstetter A, Henrich W, Rawnaq T, Hasselbeck H, Dobert TF, Hinkson L, Schwaerzler P. The misoprostol vaginal insert compared with oral misoprostol for labor induction in term pregnancies: a pair-matched case-control study. J of. Perinat Med. 2017; 10.1515/jpm-2017-0049 28672758

[13] Gornisiewicz T, Jaworowski A, Zembala-Szczerba M, Babczyk D, Huras H (2017). Analysis of intravaginal misoprostol 0.2 mg versus intracervical dinoprostone 0.5 mg doses for labor induction at term pregnancies. Ginekologia pol.

[14] Robson M, Murphy M, Byrne F (2015). Quality assurance: the 10-group classification system (Robson classification), induction of labor, and cesarean delivery. Int J Gynaecol Obstet.

[15] Pevzner L, Rayburn WF, Rumney P, Wing DA (2009). Factors predicting successful labor induction with dinoprostone and misoprostol vaginal inserts. Obstet Gynecol.

[16] Wing DA, Tran S, Paul RH (2002). Factors affecting the likelihood of successful induction after intravaginal misoprostol application for cervical ripening and labor induction. Am J Obstet Gynecol.

[17] Crane JMG, Delaney T, Butt KD, Bennett KA, Hutchens D, Young DC (2004). Predictors of successful labor induction with oral or vaginal misoprostol. J Matern Fetal Med..

[18] Chen W, Xue J, Gaudet L, Walker M, Wen SW (2015). Meta-analysis of Foley catheter plus misoprostol versus misoprostol alone for cervical ripening. Int J Gynaecol Obstet.

[19] Laughon SK, Berghella V, Reddy UM, Sundaram R, Lu Z, Hoffman MK (2014). Neonatal and maternal outcomes with prolonged second stage of labor. Obstet Gynecol.

[20] Maghoma J, Buchmann EJ (2002). Maternal and fetal risks associated with prolonged latent phase of labour. J Obstet Gynaecol.

[21] Wing DA, Miller H, Parker L, Powers BL, Rayburn WF (2011). Misoprostol vaginal insert for successful labor induction: a randomized controlled trial. Obstet Gynecol.

[22] Gregson S, Waterstone M, Norman I, Murrells TA (2005). Randomised controlled trial comparing low dose vaginal misoprostol and dinoprostone vaginal gel for inducing labour at term. BJOG.

[23] Calder AA, Loughney AD, Weir CJ, Barber JW (2008). Induction of labour in nulliparous and multiparous women: a UK, multicentre, open-label study of intravaginal misoprostol in comparison with dinoprostone. BJOG.

[24] Wing DA, Ham D, Paul RHA (1999). Comparison of orally administered misoprostol with vaginally administered misoprostol for cervical ripening and labor induction. Am J Obstet Gynecol.

[25] van Gemund N, Scherjon S, LeCessie S, van Leeuwen JHS, van Roosmalen J, Kanhai HHH. A Randomised trial comparing low dose vaginal misoprostol and dinoprostone for labour induction. BJOG 2004; 111(1):42–49. 10.1046/j.1471-0528.2003.00010.x 14687051

[26] Norwegian Institute of Public Health. Medical Birth Registry of Norway. http://statistikk.fhi.no/mfr/. Accessed 18 Oct 2016.

[27] European Medicines Agency. List of nationally authorised medical products Active substance: Misoprostol (gynaecological indication - labour induction). http://www.ema.europa.eu/docs/en_GB/document_library/Periodic_safety_update_single_assessment/2017/03/WC500223582.pdf. Accessed 02 Jan 2018.

[28] NHS Digital. NHS Maternity Statistics - England, 2013–14. https://digital.nhs.uk/catalogue/PUB16725. Accessed 19 Oct 2016.

[29] Kim LH, Cheng YW, Delaney S, Jelin AC, Caughey ABI (2010). Preeclampsia associated with an increased risk of cesarean delivery if labor is induced?. J Matern Fetal Med.

[30] Righetti-Veltema M, Conne-Perréard E, Bousquet A, Manzano J (1998). Risk factors and predictive signs of postpartum depression. J Affect Disord.

